# Feline Endocarditis Caused by Carbapenem-Resistant *Providencia rettgeri*: Clinicopathological, Echocardiographic, and Genomic Investigation

**DOI:** 10.3390/antibiotics15080778

**Published:** 2026-08-13

**Authors:** Maisa de Carvalho, Yasmin Gonçalves de Castro, Mayara Lucia Muniz Rezende, Tatyana Salarolli, Anelise Carvalho Nepomuceno, Thaynara Parente de Carvalho, Rogéria Serakides, Rodrigo Otávio Silveira Silva, Luiz Eduardo Duarte de Oliveira

**Affiliations:** School of Veterinary Medicine, Federal University of Minas Gerais (UFMG), Avenida Antonio Carlos, 6627, Belo Horizonte 30123-970, Brazil

**Keywords:** feline infective endocarditis, *bla*_NDM-1_, Col3M

## Abstract

**Background**: Bacteria of the genus *Providencia* are emerging pathogens associated with a wide range of infections, particularly in healthcare settings. In veterinary medicine, infections caused by these organisms are uncommon, and, to date, there have been no reports of carbapenem-resistant *Providencia* spp. causing infection in companion animals. **Methods and Results**: This study describes a comprehensive investigation of bacterial endocarditis caused by carbapenem-resistant *Providencia rettgeri* in a cat, including clinical, imaging, pathological, microbiological, and genomic findings. A 1.5-year-old mixed-breed female cat was admitted to a referral veterinary hospital with an extensive pelvic limb wound and a recent history of laparotomy. During hospitalization, a heart murmur was detected, and echocardiographic examination revealed vegetation-like lesion on the mitral valve. Despite intensive treatment, the patient’s clinical condition progressively deteriorated over a 15-day period, ultimately leading to euthanasia. Necropsy confirmed bacterial endocarditis with evidence of systemic septic embolization. *P. rettgeri* was isolated from blood collected from both heart ventricles and from an endocardial swab and its identity was confirmed by MALDI-ToF mass spectrometry. Antimicrobial susceptibility testing demonstrated resistance to meropenem. Whole-genome sequencing confirmed the species identification and revealed a Col3M plasmid carrying multiple antimicrobial resistance genes, including the carbapenemase gene *bla*_NDM-1_. **Discussion**: To the best of our knowledge, this is the first report of carbapenem-resistant *Providencia* spp. causing infection in a companion animal. This case highlights the emergence of *Providencia* spp. as potential veterinary pathogens capable of causing severe, invasive and fatal infections.

## 1. Introduction

Bacteria of the genus *Providencia* are Gram-negative bacilli belonging to the family Morganellaceae and the order Enterobacterales. The genus currently comprises 15 species, several of which are recognized as opportunistic human pathogens, particularly in immunocompromised or hospitalized individuals [1]. In humans, *Providencia* spp. have been implicated in infections at a variety of sites, including urinary tract infections, gastroenteritis, pneumonia, endocarditis, meningitis, and sepsis [2,3].

Infections caused by *Providencia* spp. can be treated with a variety of antimicrobial agents, including aminoglycosides, fluoroquinolones, and second- and third-generation cephalosporins. However, the increasing emergence of multidrug-resistant (MDR) strains over the past decade has substantially limited therapeutic options, leading to a greater reliance on carbapenems for the treatment of severe infections. The first carbapenem-resistant *Providencia* spp. isolate was reported in Japan in 2003 [4]. Since then, carbapenem-resistant *Providencia* spp. have been increasingly recognized as a cause of nosocomial infections worldwide, with the first report from Brazil published in 2013 [5]. Today, carbapenem-resistant Enterobacterales are classified by the World Health Organization (WHO) as Critical Priority Pathogens because of their urgent need for new therapeutic strategies to combat these organisms [6]. Among the mechanisms conferring carbapenem resistance, New Delhi metallo-β-lactamase (NDM) is one of the most frequently identified carbapenemases in *Providencia* spp. The corresponding gene, *bla*_NDM-1_, is commonly carried on conjugative plasmids, including Col3-type plasmids, facilitating horizontal gene transfer among bacterial populations [7]. Infections caused by NDM-producing bacteria represent a major global public health concern because they significantly restrict available treatment options and are associated with increased morbidity and mortality [7].

Despite their recognized importance in human medicine, infections caused by *Providencia* spp. remain rarely reported in veterinary medicine. In dogs, these organisms have been associated with urinary tract infections, cutaneous lesions, cellulitis, otitis, and gastroenteritis [8,9,10,11]. In cats, studies are even more limited, consisting primarily of isolated case reports, including pancreatolithiasis [12] and cholecystitis associated with neutrophilic cholangitis [13]. To the best of our knowledge, there are no previous reports of carbapenem-resistant *Providencia* spp. infections in companion animals or of cardiac involvement associated with this bacterial genus in dogs or cats.

Therefore, the aim of the present study was to provide a comprehensive investigation of bacterial endocarditis associated with carbapenem-resistant *P. rettgeri* in a cat, detailing the clinical, imaging, pathological, microbiological, and genomic findings. The isolate was found to harbor *bla*_NDM-1_, representing, to our knowledge, the first description of a carbapenem-resistant *Providencia* spp. infection in a companion animal and the first report of feline endocarditis associated with this bacterial genus.

## 2. Case Presentation

**Figure 1:**
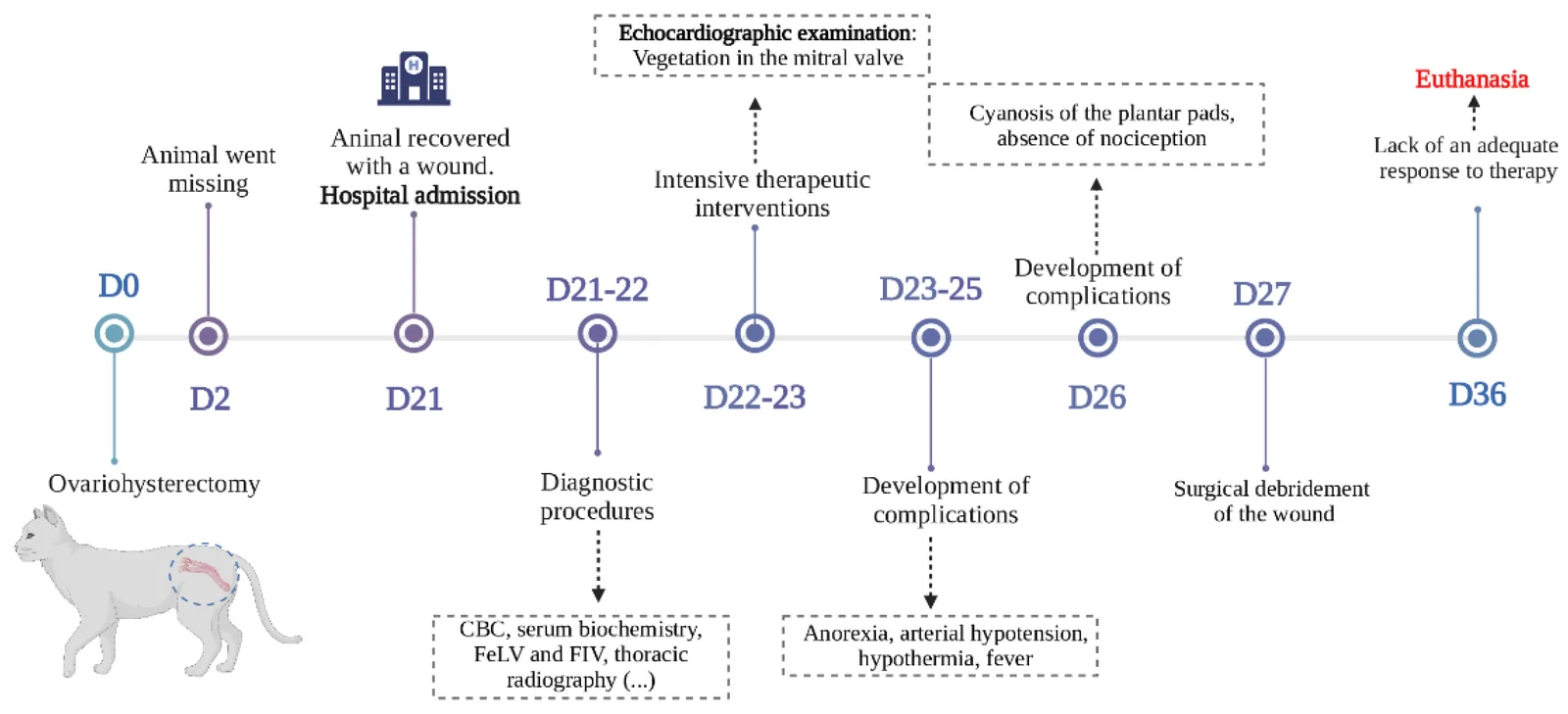
Timeline of the affected cat, from the first known surgical intervention (ovariohysterectomy) to euthanasia and the diagnosis of *Providencia rettgeri* endocarditis Created with BioRender.com.

A 1.5-year-old, 4.5 kg, mixed-breed, free-roaming female cat was presented to a referral veterinary hospital with a wound affecting the pelvic limb. During the anamnesis, the caretakers reported that the animal had undergone ovariohysterectomy 21 days prior (Figure 1). However, the patient had been missing since the second postoperative day and was recovered only on the day of presentation, already exhibiting the lesion. On physical examination, the cat had a low body condition score (BCS 2/9), depressed mentation, ataxia, absence of the menace response, approximately 8% dehydration, dyspnea and a grade II/VI systolic heart murmur auscultated over the mitral area. Additionally, an extensive wound was identified on the left pelvic limb, characterized by edema, a fetid odor, and the presence of devitalized tissue.

Ancillary diagnostics included a complete blood count, serum biochemistry, rapid tests for feline leukemia virus (FeLV) and feline immunodeficiency virus (FIV), and thoracic radiography. The patient was subsequently hospitalized for correction of fluid and electrolyte imbalances and management of the wound.

**Figure 2:**
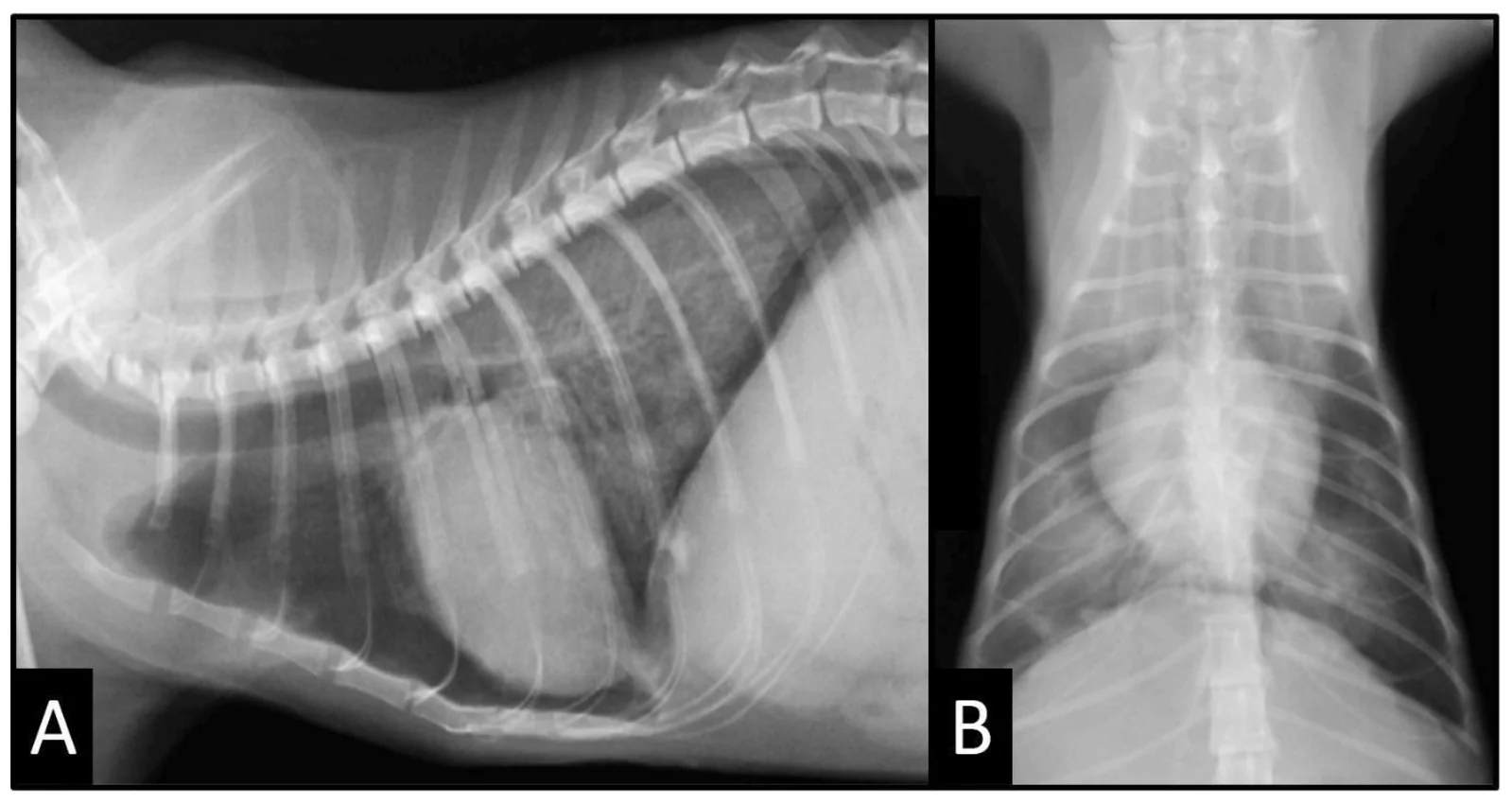
Thoracic radiographs of a 1.5-year-old mixed-breed female cat diagnosed with bacterial endocarditis caused by *Providencia* spp., demonstrating a diffuse alveolar pulmonary pattern associated with an enlarged cardiac silhouette, as well as pneumothorax and atelectasis. (**A**) Left lateral projection demonstrating a dorsal displacement of the cardiac silhouette and atelectasis, related to pneumothorax. (**B**) Ventrodorsal projection demonstrating a diffuse alveolar pulmonary pattern associated with an enlarged cardiac silhouette.

Rapid testing yielded negative results for both feline retroviruses. The complete blood count (Supplementary Table S1) revealed severe macrocytic normochromic anemia, leukocytosis due to neutrophilia with a regenerative left shift, the presence of toxic neutrophils, and thrombocytopenia. Serum biochemistry (Supplementary Table S2) demonstrated hypoproteinemia, decreased serum concentrations of creatinine and alkaline phosphatase, increased aspartate aminotransferase (AST) activity, and hyperglycemia (290 mg/dL). Thoracic radiographs showed diffuse alveolar pulmonary pattern, atelectasis, pneumothorax, and cardiomegaly (Figure 2).

Based on the clinical and laboratory findings, oxygen therapy and medical management were initiated, including furosemide (2 mg/kg, intravenous), clindamycin (5.5 mg/kg, orally, BID), ceftriaxone (40 mg/kg, intravenous, BID), tramadol hydrochloride (3 mg/kg, intravenous, BID), and dipyrone (12.5 mg/kg, intravenous, BID), in addition to blood transfusion.

During hospitalization, the patient developed anorexia, arterial hypotension (systolic blood pressure 68 mmHg), and hypothermia (35.5 °C). Continuous infusion of norepinephrine (0.5 µg/kg/min) was initiated, along with assisted feeding via a nasogastric tube and thermal support in an incubator until hemodynamic stabilization was achieved. However, over the following days, fluctuations in blood pressure and body temperature were observed, with episodes of hypotension, hypothermia, and fever.

**Figure 3:**
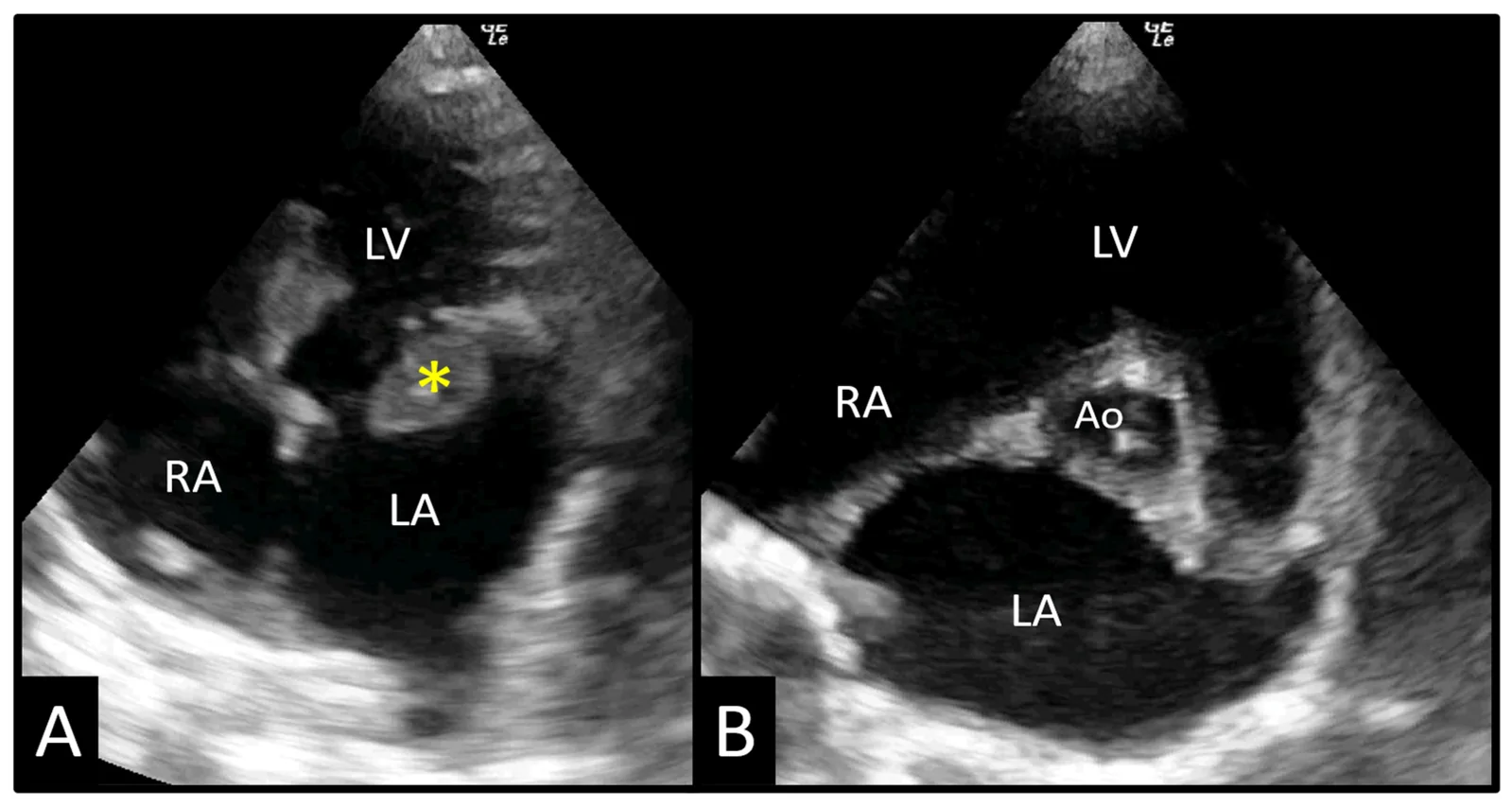
Transthoracic echocardiography of a 1.5-year-old mixed-breed female cat diagnosed with bacterial endocarditis caused by *Providencia rettgeri*. (**A**) Left parasternal apical four-chamber view demonstrating a vegetation adherent to the mitral valve (*). (**B**) Right parasternal short-axis view at the level of the heart base, demonstrating marked enlargement of the cardiac chambers. LA = left atrium; RA = right atrium; LV = left ventricle; RV = right ventricle; Ao = aorta.

Echocardiographic examination revealed a vegetation adherent to the mitral valve, associated with prolapse of the mitral valve leaflets (Figure 3). Doppler assessment and color flow mapping demonstrated mild-to-moderate mitral regurgitation (3.26 m/s; 42.63 mmHg). The tricuspid valve exhibited mild regurgitation (2.05 m/s; 16.85 mmHg). Enlargement of left and right cardiac chambers was observed, along with a restrictive left ventricular filling pattern. Based on the echocardiographic findings, differential diagnoses included bacterial endocarditis, a valvular thrombus, and a valvular neoplasm, with endocarditis considered the most consistent diagnosis given the patient’s clinical condition.

Due to marked cardiomegaly (LA/Ao ratio = 3.10), clopidogrel (5 mg/kg, oral, SID) was added to the therapeutic protocol with the aim of reducing the risk of thromboembolism. Five days after hospital admission, the patient developed cyanosis of the plantar pads, absence of nociception, and decreased temperature of the affected pelvic limb extremity. Thermographic evaluation indicated reduced perfusion of the limb. Abdominal ultrasonography was performed to investigate aortic thromboembolism; however, no thromboembolic obstruction was identified at the aortic trifurcation.

Six days after hospital admission, following hemodynamic stabilization, the patient underwent surgical debridement and primary closure of the wound. After 15 days of treatment, because of marked clinical deterioration and lack of an adequate response to therapy, the patient was euthanized and underwent for postmortem examination.

## 3. Material and Methods

### 3.1. Gross Pathology and Histopathology Investigation

A complete necropsy was performed and gross pathological examination included a systematic evaluation of all major organs and tissues. Representative tissue samples from all major organs, including the cardiac valves and any grossly altered tissues, were collected and fixed in 10% neutral-buffered formalin. Following routine processing, tissues were embedded in paraffin, sectioned at approximately 4–5 µm, and stained with hematoxylin and eosin (H&E) for histopathological examination by a veterinary pathologist. During necropsy, blood samples were collected from the left and right ventricles using sterile syringes. In addition, a swab was used to sample the valvular lesion on the mitral valve. All specimens were submitted for microbiological analysis.

### 3.2. Microbiological Isolation and Antimicrobial Susceptibility

Samples were plated onto Brain Heart Infusion agar (BHI, Oxoid, Hampshire, UK) supplemented with equine blood and onto MacConkey agar (Kasvi, Ariccia, Italy) and incubated for 48 h. Blood samples collected from left and right ventricles from the swab from the endocarditis lesion showed heavy growth of a single, homogeneous morphology, suggesting a pure culture. This colonies were subcultured in BHI agar and subjected to Matrix-Assisted Laser Desorption Ionization–Time of Flight (MALDI-ToF, Biotyper Compass version 4.1.100, BDAL library V13.0.0.2, Bruker Daltonics, Bremen, Germany) as previously described [14]. Antimicrobial susceptibility testing was performed by the disk diffusion method according to the Clinical and Laboratory Standards Institute [15]. Briefly, a bacterial suspension equivalent to a 0.5 McFarland standard was prepared from a 24 h culture and inoculated onto Mueller–Hinton agar plates (HiMedia, Mumbai, India) using sterile swabs. The following antimicrobial disks (Oxoid, Hampshire, UK) were tested: ampicillin (10 μg), cephalothin (30 μg), ceftiofur (30 μg), ceftriaxone (30 μg), meropenem (10 μg), tetracycline (30 μg), gentamicin (10 μg), enrofloxacin (5 μg), ciprofloxacin (5 μg), florfenicol (30 μg), and sulfamethoxazole/trimethoprim (23.75/1.25 μg). Plates were incubated aerobically at 37 °C for 18 h, after which inhibition zone diameters were measured and interpreted according to CLSI breakpoints [15]. *Escherichia coli* ATCC^®^ 25922 was used as the quality control strain. In addition, the minimal inhibitory concentration (MIC) of meropenem was determined using a gradient diffusion method (Etest^®^, bioMérieux, Marcy-l´Etoile, France) according to the manufacturer’s instructions. The MIC was interpreted using the CLSI breakpoints [15].

### 3.3. Whole Genome Sequencing and Comparative Genomics

One isolate recovered from the left ventricle blood sample was selected for genomic DNA extraction. The strain was plated onto BHI agar and incubated at 37 °C for 24 h. A single colony was then inoculated into 15 mL of BHI broth (Oxoid, Hampshire, UK) and incubated at 37 °C for 24 h. The resulting bacterial biomass was used for genomic DNA extraction with the Wizard Genomic DNA Purification kit (Promega, Madison, WI, USA) following the manufacturer’s instructions. Sequencing was then performed on the Illumina MiSeq platform (average output cycles of 2 × 150 bp), and the raw reads were analyzed with FastQC (Babraham Bioinformatics, Cambridge, UK), retaining only paired reads with a Phred quality score ≥30 and a minimum length of 50 nucleotides. Genome assembly was performed using SPAdes 3.5.0 in the careful mode [16], followed by gap filling and polishing with the Pilon [17]. The genome has been deposited in the NCBI database and is available online (Accession PRJNA1468575).

Bacterial species prediction was performed using a Kmer-based algorithm [18] and sequence type were determined using PubMLST (https://pubmlst.org/). ResFinder 4.5 and PlasmidFinder 2.0 [19,20] were used to identify acquired antimicrobial resistance determinants. The plasmidSPAdes [21] was used for plasmid reconstruction and Single nucleotide polymorphism (SNP) analysis was performed using CSIPhylogeny [22], with a minimum Z-score of 1.96 and using the strain DFAARGOS 1449 (Accession Number SAMN16357591) as a reference. Virulence factors were identified using the Virulence Factor Database (VFDB) [23]. For comparative analysis, publicly available genomes of *Providencia* spp. from the Bacterial and Viral Bioinformatics Resource Center (BV-BRC) and from the National Center for Biotechnology Information (NCBI) were included. The inclusion criteria were: (1) *Providencia* spp. isolates positive for *bla*_NDM-1_; (2) minimum coverage depth of 30×; (3) available metadata regarding both host and country of isolation (Supplementary Table S3). The phylogenetic tree was visualized using the iTOL v.6 online tool with midpoint rooting (https://itol.embl.de/).

## 4. Results

### 4.1. Gross Pathology and Histopathology Findings

**Figure 4:**
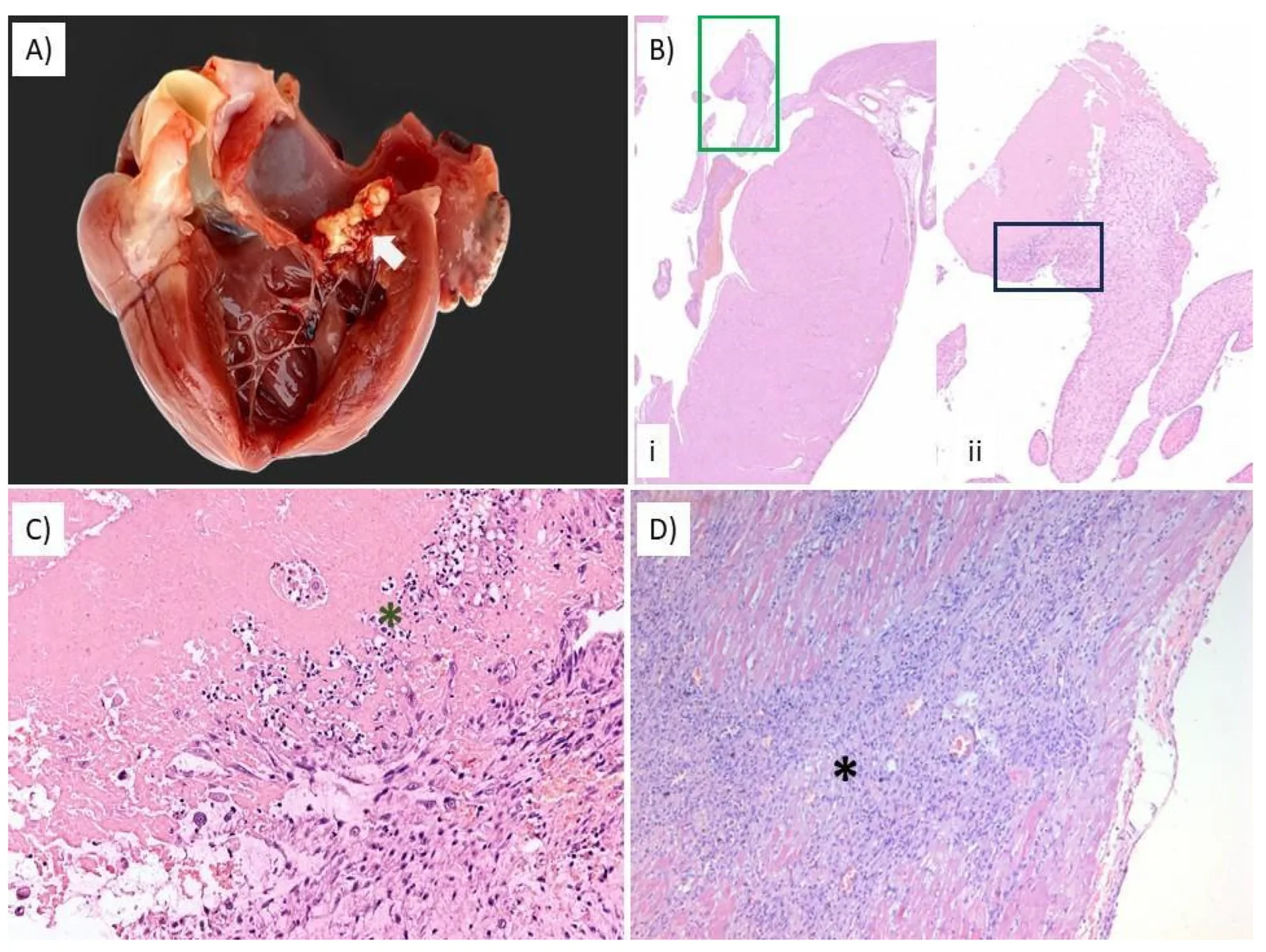
Gross and histopathological findings in the heart of a feline. (**A**) Left ventricular chamber and mitral valve exhibiting a nodular, yellowish structure adhered to the valvular surface. (**B**) Subgross images of the atrioventricular (mitral) region (i and ii) 40×, highlighting the area of endocarditis (green inset), better visualized in panel ii, 100×. (**C**) Higher magnification of the endocardial lesion (black inset from panel Bii), characterized by a neutrophilic infiltrate (green asterisk) associated with fibrin deposition and fibroblast proliferation, 200×. (**D**) Left ventricular myocardium showing a focally extensive lymphohistioplasmacytic inflammatory infiltrate with rare neutrophils, 100×. Hematoxylin and eosin (H&E) stain.

The heart exhibited mild pericardial effusion (approximately 2 mL; hydropericardium). The mitral valve was moderately thickened (0.6 cm × 0.3 cm), with an irregular surface, yellow discoloration, and a friable appearance, resulting in partial occlusion of the atrioventricular lumen, consistent with vegetative endocarditis (Figure 4A). The left atrium was moderately dilated and contained a mixed clot. Additionally, multifocal reddish nodular lesions were observed in the lungs, spleen, and kidneys, consistent with septic emboli.

Other necropsy findings included a 12 cm linear area of cutaneous discontinuity extending from the iliac fold to the femorotibiopatellar region, which had been previously sutured. The digits were reduced in size and exhibited necrosis consistent with gangrene. Postmortem radiographic evaluation of the limb ruled out fractures. Additional findings included mucosal pallor, mild hydrothorax, and marked pulmonary edema.

Histopathological examination of the heart revealed that the mitral valve contained a moderate inflammatory infiltrate composed of neutrophils and degenerate neutrophils, associated with abundant eosinophilic, amorphous to occasionally fibrillar material, containing multiple intensely basophilic, granular, and birefringent areas (necrosis and mineralization). In some regions of the valve, a lymphohistiocytic and neutrophilic infiltrate was observed, accompanied by scattered fibroblasts and erythrocytes (fibroplasia, characterized as chronic endocarditis). Within mineralized areas, small numbers of intralesional Gram-negative coccobacilli were identified (Figure 4).

Histopathological findings were consistent with a systemic infectious process characterized by septic embolic dissemination. In the spleen, multifocal areas within the red and white pulp contained intact and degenerate neutrophils, cellular debris, fibrinous material, and occasional bacteria, consistent with septic emboli. In the kidneys, a well-demarcated focus adjacent to an interlobar artery was characterized by central eosinophilic fibrillar material and erythrocytes, surrounded by macrophages, fibroblasts, hemorrhage, and lymphohistiocytic inflammation, consistent with a septic embolic lesion. In the lungs, histopathological examination revealed extensive areas of necrosis and hemorrhage associated with marked neutrophilic and lymphohistiocytic infiltration, with alveolar spaces filled with edema fluid and fibrin.

### 4.2. Microbiological and Genomic Findings

The isolates recovered from blood samples collected from the left and right ventricles, as well as from the endocardial lesion swab, were identified as *Providencia rettgeri* by MALDI-TOF mass spectrometry, with a score of 2.200. All three isolates exhibited identical antimicrobial susceptibility profiles, showing resistance to tetracycline, penicillins, first- and third-generation cephalosporins (cephalothin, ceftiofur, and ceftriaxone), and the carbapenem meropenem. In contrast, they were susceptible to gentamicin, florfenicol, and sulfamethoxazole/trimethoprim, while intermediate susceptibility was observed for the fluoroquinolones enrofloxacin and ciprofloxacin. Resistance to meropenem was further confirmed by the gradient diffusion method (Etest^®^), which demonstrated a minimum inhibitory concentration (MIC) of >32 μg/mL.

Because the isolates were recovered from different anatomical sites within the same animal and displayed identical antimicrobial susceptibility profiles, they were considered likely to be clonal. Therefore, a single isolate recovered from the blood collected from the left ventricle was selected for whole-genome sequencing.

The isolate was successfully sequenced, yielding an average genome coverage of 41.8x, assembled into 71 contigs with an N50 of 230,251 bp and a total genome size of 4.94 Mb. No evidence of contamination was detected.

**Figure 5:**
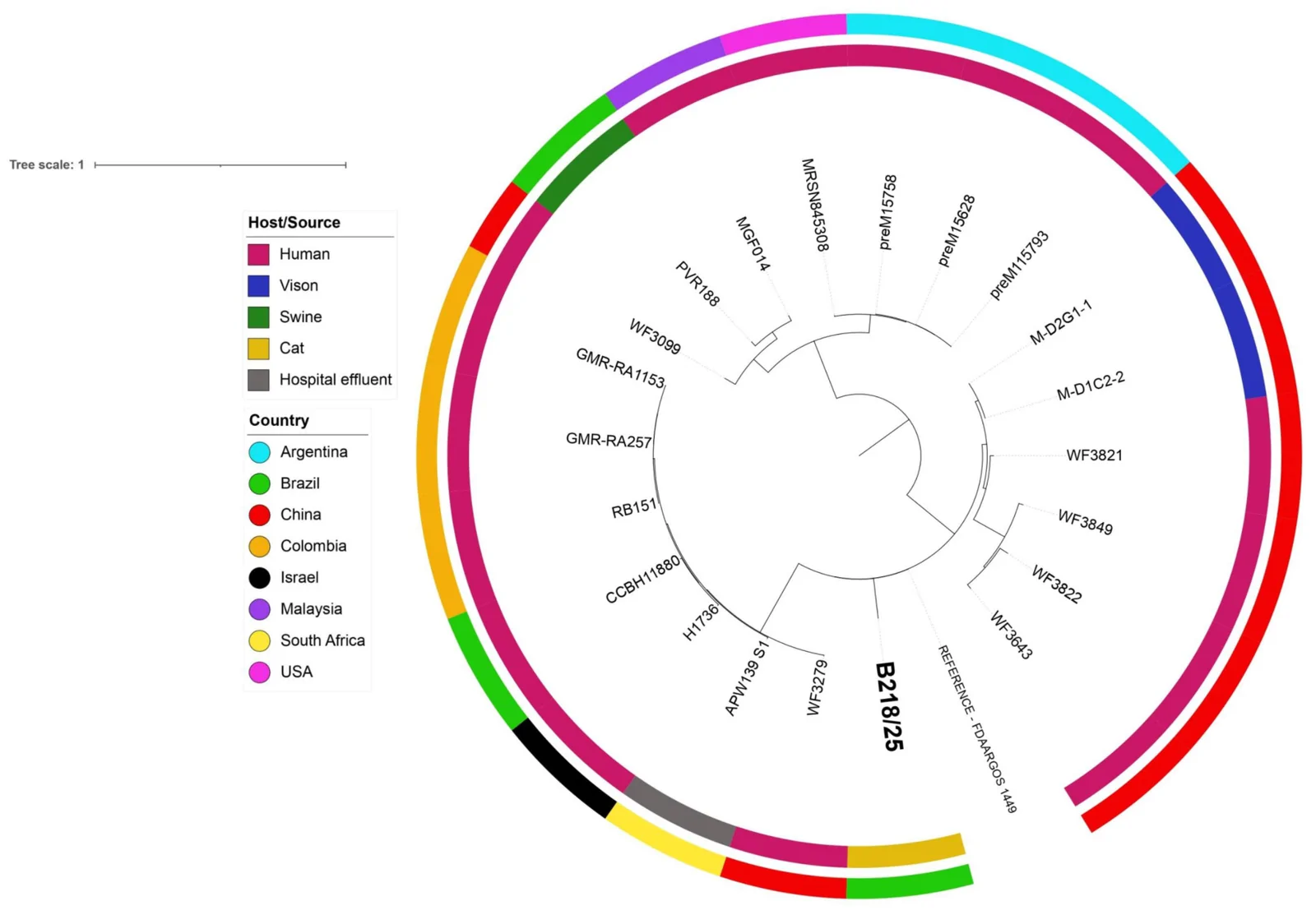
Single-nucleotide polymorphism (SNP) analysis showing the genetic relationship of the feline *Providencia* spp. isolate (B218/25) with *bla*_NDM-1_-positive strains from different hosts, including humans. The tree visualization was generated using iTOL v.6 online applying midpoint rooting.

Species prediction based on a Kmer algorithm identified the isolate as *Providencia rettgeri* as the likely species, corroborating the results seen in the MALDI-ToF. Multilocus sequence typing (MLST) classified the isolate as a novel sequence type, which was assigned ST389. Virulence factor screening identified genes associated with lipopolysaccharide biosynthesis (*kdsA*) and flagellar structure and motility (*fliC*, *fliG*, *fliN*, and *fliP*). The analysis of acquired antimicrobial resistance determinants identified the presence of the *bla*_NDM-1_ gene, encoding a carbapenemase that confers resistance to nearly all beta-lactam antibiotics, including carbapenems. In addition, genes associated with resistance to fluorquinolones (*qnrD1*), macrolides and streptogramins (*msr(E)* and *mph(E)*), tetracyclines (*tetB*) and aminoglycosides (*aph(3’)-VI*) were detected. The reconstructed plasmid was predicted to harbor *bla*_NDM-1_, *msr(E)*, *mph(E)* and *aph(3’)-VI*. Single nucleotide polymorphism (SNP) analysis demonstrated that the isolate was genetically distinct from previously reported *bla*_NDM-1_-positive *Providencia rettgeri* (Figure 5), differing from at least >4000 SNPs (Supplementary Table S4).

## 5. Discussion

This is the first report of a carbapenem-resistant *Providencia* spp. infection in a cat, as well as the first description of infective endocarditis caused by *Providencia* spp. in an animal. Infective endocarditis is rarely diagnosed in this species, with an estimated prevalence of approximately 0.007% of clinical cases, although underdiagnosis is likely [24]. There is no consistent evidence of sex or breed predisposition, and the disease is more commonly described in middle-aged to older animals, in contrast to the present report [24]. The etiology is predominantly bacterial, typically involving opportunistic microorganisms capable of colonizing the endocardium following episodes of bacteremia [25]. Reported etiologic agents in cats include bacteria from genera *Enterococcus* [26,27,28], *Bartonella henselae* [24], and *Streptococcus suis* [29], highlighting the diversity of opportunistic pathogens associated with this disease.

The genus *Providencia* has emerged as an important opportunistic pathogen in human nosocomial infections, often linked to multidrug resistance and high morbidity [1,3,30]. In veterinary medicine, the role of *Providencia* species remains poorly defined [8]. Accordingly, the present report expands the spectrum of pathogens associated with feline infective endocarditis and suggests that *Providencia* spp. should be considered among potential agents in cardiovascular infections in companion animals.

In the present case, the source of infection leading to endocarditis could not be definitively established. It can be hypothesized that the recent ovariohysterectomy, together with the presence of an infected cutaneous wound, may have contributed to the development of bacteremia and subsequent valvular infection. However, because cultures were not obtained from these potential sites of infection, this hypothesis could not be confirmed. In fact, the primary source of infection remains unidentified in most cases of bacterial endocarditis in cats [24]. Nevertheless, cutaneous abscesses, suppurative dermatitis, ovariohysterectomy, and sepsis have all been recognized as potential predisposing factors [24].

The clinical signs observed in the present case were consistent with those reported for feline infective endocarditis, reflecting both the systemic inflammatory state and the associated cardiovascular involvement. Notable findings included lethargy, hyporexia, weight loss, dehydration, and a cardiac murmur [24,26,29,31]. Respiratory distress was also observed and represents a common manifestation of feline infective endocarditis. In contrast to dogs, cats frequently develop congestive heart failure, resulting in secondary respiratory compromise [24,25,26,29,31]. Additionally, the multifocal pulmonary lesions suggestive of septic embolization may have further contributed to respiratory dysfunction through parenchymal bacterial infection.

The diagnosis of infective endocarditis remains challenging and is often established only during post-mortem examination. In an effort to improve antemortem diagnosis, Palerme et al. [24] proposed an adaptation of the Duke criteria, originally developed for humans and later applied in dogs [32,33], to estimate the probability of infective endocarditis in cats. In the present case, the echocardiographic identification of a structure consistent with a vegetation constitutes a major criterion, while fever, vascular phenomena, mitral insufficiency, and signs of sepsis represent minor criteria, supporting a high diagnostic probability. Microbiological techniques, particularly blood cultures, are essential for definitive etiological identification [24]. Unfortunately, in vivo blood cultures were not available at our hospital at the time, limiting the antemortem confirmation of the diagnosis and representing an important diagnostic constraint. 

Another useful diagnostic technique in this case was thoracic radiography, which revealed cardiomegaly and pneumothorax. Thoracic radiographs are valuable for screening feline infective endocarditis and identifying secondary changes. Cardiomegaly may or may not be present [26,29], and a range of pulmonary patterns has been described, including diffuse interstitial [29], bronchointerstitial [28], and bronchial [25] patterns. It is also important to note that echocardiographic assessment was pivotal in establishing the presumptive diagnosis of endocarditis in this case, as it enabled visualization of characteristic findings of valvular vegetation and valvular dysfunction, corroborating previous reports [24,25,26,28,29]. Taken together, the clinical signs consistent with infective endocarditis, along with the echocardiographic and radiographic findings, provided strong evidence supporting the diagnosis of infective endocarditis. This diagnosis was subsequently confirmed by postmortem examination and bacterial culture of the valvular lesion.

The isolation of *P. rettegeri* in the present work was unexpected: although the genus *Providencia* is well-recognized cause of a wide range of human infections, including endocarditis, reports of infections in animals remain scarce [3,8,9,10,11]. The antimicrobial resistance was also noteworthy, as the isolate exhibited resistance to tetracycline and several beta-lactams, including carbapenems. This finding prompted whole-genome sequencing to characterize the genetic basis of the multidrug resistance phenotype.

Interestingly, whole-genome sequencing showed excellent agreement with the phenotypic antimicrobial susceptibility results: the presence of *bla*_NDM-1_ explained the resistance to carbapenems, whereas the resistance to tetracyclines was consistent with the detection of *tet(B)*, which encodes an efflux pump [34]. Also, the reduced susceptibility to fluoroquinolones was supported by the presence of *qnrD1*, a gene that protects DNA gyrase and confers low-level resistance to this antimicrobial class [35]. In addition, whole-genome sequencing identified resistance genes for antimicrobial classes that were not evaluated phenotypically, including *msr(E)* and *mph(E)*, associated with resistance to macrolides and streptogramins, and *aph(3)-VI*, which confers resistance to aminoglycosides, particularly amikacin [36,37].

Over the past decade, the emergence and global dissemination of carbapenem-resistant *Providencia* spp. have become a major global public health concern [2]. In Brazil, Valiatti et al. (2024) [38] reported the occurrence of carbapenem-resistant *Providencia rettgeri* in a food-producing animal. In contrast, a large-scale study evaluating more than 7000 canine and feline samples failed to identify carbapenem-resistant *Providencia* spp. [39]. Taken together, these findings indicate that, to the best of our knowledge, carbapenem-resistant *Providencia* spp. have not previously been reported as causes of infection in companion animals.

In the present report, genomic analysis identified the *bla*_NDM-1_ gene together with resistance determinants associated with three additional antimicrobial classes. These genes were all predicted to be located on a Col3m plasmid, which has been implicated in the dissemination of antimicrobial resistance genes among clinically relevant Gram-negative bacteria, including nosocomial pathogens such as *Morganella morganii* and *Proteus mirabilis*, highlighting their potential role in the horizontal spread of carbapenem resistance within healthcare-associated bacterial populations [7,40,41]. This finding adds to the growing concern that companion animals may act as reservoirs and potential sources of antimicrobial-resistant bacteria and resistance genes for humans, as recently reported [42,43].

In addition to the antimicrobial resistance determinants, we screened the *P. rettgeri* genome for putative virulence-associated genes. The analysis identified a limited number of genes: one associated with lipopolysaccharide biosynthesis (*kdsA*) and several involved in flagellar structure and motility (*fliC*, *fliG*, *fliN*, and *fliP*). The *kdsA* gene encodes an enzyme involved in components of the LPS inner core, which may influence bacterial survival and host interactions [44]. The *fli* genes encode components of the bacterial flagellum, a structure associated with motility, adhesion, and biofilm formation, all of which may facilitate colonization and persistence during infection [45]. These genes are common and well-conserved in several Gram-negative bacteria commonly reported in endocarditis [25,44,45], and their presence is consistent with the pathogenic potential of *P. rettgeri*.

The identification of *P. rettgeri* in the present case is noteworthy for two reasons. First, to the best of our knowledge, there are no previous reports of *Providencia* spp.-associated endocarditis in dogs or cats. Second, this is the first description of an infection caused by a carbapenem-resistant *Providencia* spp. in an animal. Based on these findings, we initially hypothesized that the isolate might be closely related to carbapenem-resistant *Providencia* strains previously reported in human infections. However, the genomic analyses did not support this hypothesis. The isolate was assigned to a novel sequence type (ST389) by MLST and formed a distinct lineage in the SNP analysis, differing by more than 4000 SNPs from the available genomes included in the comparison. Therefore, the origin of this isolate remains unknown. A similar observation was recently reported by Zanon et al. (2025) [42], who described one carbapenem-resistant *Acinetobacter baumannii* isolate infecting a dog that were also genetically unrelated to previously characterized human strains. One possible explanation is that resistance plasmids, such as those carrying *bla*_NDM-1_ in companion animals, particularly under antimicrobial selective pressure. However, the available evidence is insufficient to determine the evolutionary origin of these strains. Further genomic surveillance of multidrug-resistant bacteria from companion animals will be essential to clarify their emergence and potential role in the epidemiology of antimicrobial resistance.

The detection of a *bla*_NDM_-positive *Providencia* sp. in a cat is of significant public health concern and highlights the role of companion animals as both susceptible hosts and potential reservoirs of antimicrobial-resistant bacteria. Notably, carbapenem-resistant Enterobacterales are classified by the WHO as Critical Priority Pathogens because of the increased morbidity and mortality associated with these organisms [6,7]. The present report reinforces the need for a One Health approach to mitigate the impact of antimicrobial resistance on both animal and human health.

The present report has some limitations. First, the source of infection leading to infective endocarditis could not confirmed because bacterial cultures were not obtained from the two known potential sites of infection. Second, blood cultures were not performed during hospitalization. Although the sensitivity of blood cultures in cases of infective endocarditis is often limited, a positive result could have guided targeted antimicrobial therapy and, consequently, may have increased the chances of survival in this case. Finally, the plasmid reconstruction from the present study was based exclusively on short-read sequencing data. Although the analysis consistently identified a Col3M plasmid carrying *bla*_NDM-1_ gene together with other antimicrobial resistance determinants, short-read assemblies cannot always resolve complete plasmid structures [46]. Therefore, the exact genetic context of the resistance determinants should be interpreted with caution. Long-read sequencing or a hybrid sequencing approach would be necessary to fully characterize the plasmid structure and gene content. Nevertheless, our findings are consistent with previous reports describing *bla*_NDM_-carrying Col3M plasmids. Together with the carbapenem-resistant phenotype, these results provide strong evidence for the presence of a plasmid harboring this clinically important carbapenemase gene.

## 6. Conclusions

This is the first report of *Providencia* spp. causing endocarditis in an animal. In addition, this is the first description of carbapenem-resistant *Providencia* spp. causing infection in a companion animal. The present investigation suggests that, similar to what has been observed in humans, *Providencia* spp. should be considered emerging pathogens in veterinary medicine, capable of causing invasive and fatal infections.

## Data Availability

The original contributions presented in this study are included in the article/Supplementary Material. Further inquiries can be directed to the corresponding authors.

## Supplementary Materials

The following supporting information can be downloaded at: https://www.mdpi.com/article/10.3390/antibiotics15080778/s1, Table S1: Serial erythrogram and leukogram results of a 1.5-year-old mixed-breed female cat diagnosed with bacterial endocarditis caused by *Providencia* spp., obtained at admission and during follow-up (24 h to 14 days), compared with reference intervals established for the species; Table S2: Serial serum biochemical profile of a 1.5-year-old mixed-breed female cat diagnosed with bacterial endocarditis caused by *Providencia* spp., obtained at admission and during follow-up (4, 6, and 14 days), compared with reference intervals established for the species; Table S3: *Providencia* spp. genomes included for comparison; Table S4: SNP Matrix.

## Abbreviations

The following abbreviations are used in this manuscript:

MLST	Multilocus sequence typing
FeLV	Feline leukemia virus
FIV	Feline immunodeficiency virus
NDM	New Delhi Metallo-β-Lactamase
MALDI-ToF	Matrix-Assisted Laser Desorption/Ionization Time-of-Flight Mass Spectrometry
BID	*bis in die* or twice a day
