# Supplementary material for: Feline Endocarditis Caused by Carbapenem-Resistant Providencia rettgeri: Clinicopathological, Echocardiographic, and Genomic Investigation
Source: Antibiotics (Basel). 2026 Aug 13;15(8):778. doi: 10.3390/antibiotics15080778 (PMC13509409; doi:10.3390/antibiotics15080778)
Supplement: Supplementary file 1 [file antibiotics-15-00778-s001.zip › Table S1_CBC.pdf]

Table S1: Serial erythrogram and leukogram results of a 1.5-year-old mixed-breed female cat diagnosed with bacterial endocarditis caused by *Providencia* spp., obtained at admission and during follow-up (24 hours to 14 days), compared with reference intervals established for the species.

**Red blood cell**	**Admission**	**24 hours**	**48 hours**	**4 days**	**6 days**	**8 days**	**10 days**	**14 days**	**Reference**
RBC (10^6/ul)	3.68*	3.31*	5.66	5.73	5.16*	4.66*	5.41*	4.85*	5.5 to 10.0
Hgb (g/dL)	4.6*	4*	7.39*	7.37*	6.94*	6.33*	7.48*	6.4*	8 to 15
Hct (%)	15*	11*	23*	23*	21*	19*	21*	20.6*	24 to 45
MCV (fl)	40.8**	33.2*	40.6	40.1	40.7	40.8	38.8*	42.5	39 to 55
MCH (pg)	12.5*	12.1*	13.1	12.9	13.4	13.6	13.8	13.2	12.6 to 17.6
MCHC (g/dL)	30.7	36.4**	32.1	32	33	33.3	35.6	31.2	30 to 36
RDW (%)	25.1**	14.3*	16.1*	16.1*	16.1*	15*	15*	30.7**	18 to 22
**White blood cell differential**									
WBC	38.850**	87.837**	42.713**	28.600**	36.750**	26.600**	55.455**	49.700**	5.500 to 19.500
Neutrophils	30.303**	69.392**	36.734**	23.738**	32.340**	22.078**	42.658**	38.269**	2.500 to 12.500
Band neutrophils	1.166**	10.541**	1.708**	572**	368**	798**	6.932**	497**	0 to 300
Lymphocytes	5.439	5.270	2.990	2.860	2.205	2.660	2.666	8.449**	1.500 to 7.000
Eosinophils	388	878	427	572	735	532	0	994	0 to 1.500
Basophils	0	878**	0	286**	0	266**	533**	497**	rare
Monocytes	1.554**	878**	854**	572	1.102**	266	2.666**	994**	100 to 850
**Platelets**	52.000*	39.000	75.000	72.000	171.000	132.000	151.000	201.000	300.000 to 500.000

MCV = mean corpuscular volume; MCH = mean corpuscular hemoglobin; MCHC = mean corpuscular hemoglobin concentration; g/dL = grams per deciliter; % = percent; fL = femtoliter; pg = picograms; mg/dL = milligrams per deciliter. *Values below the reference range established for the species. **Values above the reference range established for the specie
