# Supplementary material for: Feline Endocarditis Caused by Carbapenem-Resistant Providencia rettgeri: Clinicopathological, Echocardiographic, and Genomic Investigation
Source: Antibiotics (Basel). 2026 Aug 13;15(8):778. doi: 10.3390/antibiotics15080778 (PMC13509409; doi:10.3390/antibiotics15080778)
Supplement: Supplementary file 1 [file antibiotics-15-00778-s001.zip › Table S2_SB.pdf]

Table S2. Serial serum biochemical profile of a 1.5-year-old mixed-breed female cat diagnosed with bacterial endocarditis caused by *Providencia* spp., obtained at admission and during follow-up (4, 6, and 14 days), compared with reference intervals established for the species.

Analyte	Admission	4 days	6 days	14 days	Reference
Urea (mg/dl)	59.79	18.87	14.90	27.92	10 to 60
Creatinine (mg/dl)	0.68*	0.60*	0.33*	0.57*	0.8 to 1.8
ALT/TGP (U/L)	76.2	77.20	51.30	28.50	6 to 83
AST/TGO (U/L)	115,4**	82.50	40.00	26.80	6 to 83
ALP (mg/dl)	18.62*	27.05	21.77*	18.66*	25 to 93
GGT (U/L)	-	-	3.30	9.45**	1.3 to 5.3
Glucose (mg/dl)	290**	124.70	141.70**	154.60**	73 to 134
Total protein (g/dl)	4.84*	5.24*	6.15	7.59	5.4 to 7.8
Albumin (g/dl)	2.08*	2.14	2.38	2.03*	2.1 to 3.3
Globulin (g/dl)	2.76*	3.10*	3.77	5.56**	3.3 to 4.5

ALT: alanine aminotransferase; AST: aspartate aminotransferase; ALP: alkaline phosphatase; GGT: gamma-glutamyl transferase; mg/dl: milligrams per deciliter; g/dL: grams per deciliter; U/L: units per liter. *Values below the reference interval; **Values above the reference interval.
